# Nano-Size Characterization and Antifungal Evaluation of Essential Oil Molecules-Loaded Nanoliposomes

**DOI:** 10.3390/molecules27175728

**Published:** 2022-09-05

**Authors:** Katya M. Aguilar-Pérez, Dora I. Medina, Roberto Parra-Saldívar, Hafiz M. N. Iqbal

**Affiliations:** 1 School of Engineering and Sciences, Tecnologico de Monterrey, Atizapan de Zaragoza 52926, Estado de Mexico, Mexico; 2 Institute of Advanced Materials for Sustainable Manufacturing, Tecnologico de Monterrey, Monterrey 64849, Nuevo León, Mexico; 3 School of Engineering and Sciences, Tecnologico de Monterrey, Monterrey 64849, Nuevo León, Mexico

**Keywords:** EO molecules, nanoliposomes, nano-size characterization, trichophyton rubrum, antifungal attributes

## Abstract

Nanoliposomes, bilayer vesicles at the nanoscale, are becoming popular because of their safety, patient compliance, high entrapment efficiency, and prompt action. Several notable biological activities of natural essential oils (EOs), including fungal inhibition, are of supreme interest. As developed, multi-compositional nanoliposomes loaded with various concentrations of clove essential oil (CEO) and tea tree oil (TTO) were thoroughly characterized to gain insight into their nano-size distribution. The present work also aimed to reconnoiter the sustainable synthesis conditions to estimate the efficacy of EOs in bulk and EO-loaded nanoliposomes with multi-functional entities. Following a detailed nano-size characterization of in-house fabricated EO-loaded nanoliposomes, the antifungal efficacy was tested by executing the mycelial growth inhibition (MGI) test using *Trichophyton rubrum* fungi as a test model. The dynamic light scattering (DLS) profile of as-fabricated EO-loaded nanoliposomes revealed the mean size, polydispersity index (PdI), and zeta potential values as 37.12 ± 1.23 nm, 0.377 ± 0.007, and −36.94 ± 0.36 mV, respectively. The sphere-shaped morphology of CEO and TTO-loaded nanoliposomes was confirmed by a scanning electron microscope (SEM). The existence of characteristic functional bands in all tested counterparts was demonstrated by attenuated total reflection-Fourier transform infrared (ATR-FTIR) spectroscopy. Compared to TTO-loaded nanoliposomes, the CEO-loaded nanoliposomes exhibited a maximum entrapment efficacy of 91.57 ± 2.5%. The CEO-loaded nanoliposome fraction, prepared using 1.5 µL/mL concentration, showed the highest MGI of 98.4 ± 0.87% tested against *T. rubrum* strains compared to the rest of the formulations.

## 1. Introduction—Problem and Opportunities

Nanoliposomes are one of the most cost-effective nanocarriers utilized in the pharmaceutical sector because of their facile preparation, high entrapment efficiency, and raw material availability to fabricate them [1]. Nanoliposomes are vesicles with an average particle size of around 20 to 150 nm, composed of phospholipids that can entrap hydrophobic and hydrophilic drugs in their structure. Many natural constituents, such as soybean lecithin (SBL), egg yolk, sunflower, etc., can be used to nanofabricate bilayer structures as nanoliposomes, which increases their biocompatibility and entails a synergistic effect because it is possible to entrap hydrophobic and hydrophilic compounds at the same time [2,3]. Skin fungal infections are a significant concern worldwide, affecting about 300 million people each year globally [4]. The occurrence of cutaneous fungal infections is associated with dermatophytes [5]. Dermatophytes are groups of keratinophilic fungi that colonize humans’ skin, nails, and hair [6]. Among dermatophytes, *T. rubrum* is one of the most prevalent species associated with 80–90% of fungal skin infections such as onychomycosis and tinea pedis (athlete’s foot) [7]. Nowadays, conventional treatments of fungal infections include local administration such as topical delivery (e.g., creams, lotions, and gels) or systemic administration through oral routes (pills, tablets, etc.) [8,9]. Topical antifungal drugs related to azoles (e.g., miconazole, ketoconazole, and terconazole) have shown increased burning, itching, or irritation when applied to the skin [10]. Moreover, the adverse effects of systemic administration include unnecessary tiredness, loss of appetite, stomach upset, and fever [11].

For these reasons, the development of novel drug delivery systems based on nanotechnology has gained attention worldwide to reduce side effects and enhance the therapeutic efficacy of antifungal drugs [12,13]. Among these systems, nanoliposomes signify the most promising drug delivery agents to treat fungal infections [2,10,13,14]. The above-mentioned properties help to entrap conventional drugs into nanoliposomes to develop the safest, biocompatible, eco-friendly, and low-toxicity treatments [15,16]. Highlighting these considerations, the use of essential oils (EOs) for pharmaceutical purposes has received particular attention, which mainly owes to their bioactive entities and characteristics, including anti-inflammatory, anti-cancer, analgesic, anti-oxidant, and anti-microbial (e.g., anti-fungal) effects. EOs are natural volatile compounds produced mainly from plant raw materials or herbs (e.g., clove, tea tree, turmeric, garlic, ginger, cassia, geranium, sunflower, etc.) [17,18,19]. The bioactivities mentioned above are largely attributed to the presence of terpenes and terpenoid entities in their chemical composition profile. Among them, terpenoids are considered to be biochemically improved terpenes. For instance, enzyme-assisted modification can either incorporate oxygen molecules or interchange/eliminate some surface-pendant functional groups, such as the methyl group. Thus, the oxygen-containing terpenes are called terpenoids of biological interest [18]. The denomination of EOs was first coined by Paracelsus von Hohenheim, a Swiss reformer of medicine in the 16^th^ century. The International Organization for Standardization (ISO) (ISO/D1S9235.2) has also defined EOs as a product made by distillation with either water or steam, or by mechanical dispensation or by dry distillation of natural materials, such as plants [20].

Tea tree oil (TTO) has largely been extracted from *Melaleuca alternifolia* leaves. *M. alternifolia* plants belong to the *Myrtacea*, or *Myrtle*, family that has long been used in folk medicine to treat inflammatory and infectious problems. Based on the literature evidence, several chemically and biologically active compounds make up a major composition of TTO. Some example concentrations in percent (%) include 64.1% 1,8-cineole, 53.7% terpinen-4-ol, 45.6% terpinolene, 35.3% p-cymene, 23.2% ץ-terpinene, 12.9% α-terpinene, and 11.8% α-terpineol [21]. Thus, due to the presence of the above-mentioned wide range of functional compounds, TTO has shown a broad spectrum of multi-functional activities, including antibacterial [22], antifungal [23], anti-inflammatory [24], antioxidant, anti-tumor, and immune regulation effects [25]. Like TTO, clove essential oil (CEO) is extracted from *Syzygium aromaticum*. *S. aromaticum*, a tropical evergreen tree, which also belongs to the *Myrtaceae* family of medicinal value. In CEO composition, eugenols, β-caryophyllene, eugenyl acetate, α-humulene, and caryophyllene oxide have been documented as major chemical elements [26]. The medical values and pharmacological characteristics of CEO include anti-inflammatory, anti-cancer, analgesic, anti-oxidant, and anti-microbial (e.g., anti-bacterial, anti-fungal, anti-viral), insecticidal, anti-mutagenic, and hepatoprotective effects [27].

Notwithstanding the potential of EOs, their medicinal and pharmacological properties still use extant limitations, such as low miscibility in water and high volatilization rate, which results in less bioavailability and low stability [28]. Aiming to tackle these limitations, nanoencapsulation offers a unique solution to encapsulate highly volatile and unstable biomolecules, such as EOs, in nanoliposomes. In this way, the nanoencapsulation protects them from rapid volatilization, avoids degradation, improves their efficiency, and stuns their susceptibility [29]. Encapsulation also increases the EOs’ solubility, induces durability for thermal processing, and enhances the shelf life. Thus, it enhances their applicability and improves pharmacological activity [30]. In this regard, it is possible to avoid the acquaintance and deprivation of EOs and their bioactive entities by generating physical barriers and facilitating their controlled release [31,32,33,34,35].

Considering the above critiques and rationale behind the multi-functional characteristics of nanoliposomes, this work is aimed at (i) in-house fabrication of multi-compositional nanoliposomes comprising various CEO and TTO concentrations, (ii) analytical and imaging characterization of TTO and CEO-loaded nanoliposomes to evaluate the efficacy of EOs in bulk and CEO/TTO loaded nanoliposomes, (iii) nano-size evaluation of particle size, PdI, surface charge, and morphology, and (iv) anti-fungal potential of EOs and CEO/TTO-loaded nanoliposomes against *T. rubrum.*

## 2. Results and Discussion

### 2.1. Nano-Size Distribution of CEO and TTO-Loaded Nanoliposomes

The average particle size distribution, PdI, and zeta potential of as-fabricated fractions, i.e., CEO and TTO-loaded nanoliposomes, were recorded on day 1, day 7, and day 30 after fabrication. Among all prepared fractions, F2 CEO and F3 CEO displayed the lowest particle size distribution. The average particle size distribution of samples, i.e., F2 CEO and F3 CEO, was in the range of 71.43 ± 1.42 nm and 72.88 ± 0.33 nm, respectively, after day 1. Furthermore, after day 7, a drop in the size of the F2 CEO, i.e., 68.26 ± 0.49 nm was found, while it was 71.45 ± 0.53 nm for the F3 CEO. As compared to day 1 samples, the day 30 samples displayed a larger particle size for all formulations of CEO and TTO-loaded nanoliposomes. The characteristics, such as smaller particle size, higher surface area, and strong interface, among particles result in agglomeration [36]. Thus, the average particle size distribution increases with a more extended storage period as observed herein. Several aspects have been tested as primary reasons that ultimately influence the firmness of nanoliposome systems. For instance, (1) any possible degradation of nanoliposome’s components due to the hydrolysis and oxidation type reaction during the preparation or storage, (2) the physical stability of the nanoliposome fractions can be altered due to any possible chemical changes in the layer-forming molecules, such as the nanoliposome structure, is affected as phospholipids lose one of the acyl-chains, and (3) the agglomeration phenomenon also contributes to altering the physiological appearance of the nanoliposomes by varying the lipid layer arrangements. Thus, in the case of agglomeration, the average size distribution of target nanoliposomes should increase, as evident from the results obtained herein. The storage stability of nanoliposomes in terms of size may be increased by using chemical-grade phospholipids [37].

PdI signifies the dispersal of population sizes that characteristically range from 0.0 to 1.0. As testified in the literature, PdI values of 0.2 and/or below are suitable for polymer-based nanoconstructs [38,39]. However, among the drug delivery systems based on lipid-based nano-carriers, e.g., nanoliposome-based active fractions, the PdI values range from 0.3 and/or lower to display a consistent population of phospholipid vesicles [40]. The recorded PdI values of CEO-loaded nanoliposome fractions were 0.296 ± 0.008 up to 0.548 ± 0.008. After day 7 of storage, the F3 CEO nanoliposome formulation showed a PdI of 0.296 ± 0.008, whereas the formulation F5 CEO after day 30 of storage displayed an average PdI of 0.548 ± 0.008. As noted, after day 1 and day 7 of storage, the formulations prepared using lower respective EO (CEO/TTO) concentrations displayed lower PdI values. For comparison purposes, the empty vesicle was used as a control sample. The mono-disperse samples have smaller sizes and thus show a lower PdI value. However, in some cases, small particle size values reached PdI values higher than 0.3, indicating partial homogeneous suspension. Concerning the CEO concentration and its influence on the particle size distribution, the particle size of F1 CEO was 74.67 ± 0.81 nm on day 1, which was increased to 86.0 ± 41.43 nm and 97.49 ± 1.48 nm on day 7 and day 30, respectively. Overall, after 30 days of storage, F1 fraction was prepared using a 6.2 mM concentration comprising 97.49 ± 1.48 nm particle size, and F4 was prepared using a 2.5 mM concentration containing the average particle size of 95.98 ± 0.61 nm, which got the least particle size distribution as compared to F6. The involvement of the cavitation procedure allows for interplay between lipids and surfactants at the interface [41].

Thus, the particle size increases at high CEO concentrations. There could be an interaction between the CEO, a hydrophobic agent, and the lecithin bilayer’s acyl chains, which is responsible for altering the acyl chain order. Since CEO is freely positioned in the lecithin structure, which is also responsible for the nanoliposome development, it effectively contributes to the thickness of the nanoliposomes. Whereas a decrease in particle size will be observed [42] if the functional material attains better encapsulation. Our findings indicate that at higher concentrations of EO, a more uniform particle size is achieved compared to lower concentrations of EO and the control sample (empty nanoliposomes). The bulk of nanoliposomes would be larger in the case of the F6 sample. The mixture of lipids, surfactant, and sterol remained alone without adding any EO [43,44]. The PdI values were less than 0.5 in all samples, which indicates a decent particle distribution. The average particle size and PdI values for all CEO nanoliposomes are presented in Table 1.

The average particle size distribution for TTO-loaded nanoliposomes was smaller than CEO-loaded nanoliposomes at higher concentrations of EOs. Similarly, in the case of the F1 TTO fraction, the decrease was observed in the particle size distribution from 44.84 ± 1.98 nm to 37.27 ± 0.46 nm after day 7. This can be correlated to the segmentation of TTO from the bilayer dispersion medium to sustain the equilibrium [42]. However, after day 30, there was an enlargement in the particle size up to 88.54 ± 0.19 nm, which remained in the nano-range. After day 7 and day 30, the control vesicles’ average particle size displayed a large particle size compared to the TTO-loaded formulations. There was a decrease in the particle size in F2 TTO after day 30 and F3 TTO after day 7 of storage, respectively. The average particle sizes and PdI values of TTO-loaded nanoliposomes are presented in Table 2.

### 2.2. Zeta Potential Values for CEO and TTO-Loaded Nanoliposomes

Zeta potential evaluation assistsed in determining the characteristic surface charge of a suspension [40]. In nanoliposomes, the lipid component participates in the electrophoretic mobility of vesicles. Correspondingly, the functional lipophilic material shields the charge of coating phospholipid [45]. The zeta potential profile of CEO-loaded nanoliposomes revealed values ranging −30.39 ± 0.79 mV, being higher than F6. The detailed summary of zeta potential values of all test formulations after day 1, day 7, and day 30 of storage is summarized in Table 3. The representative values in minus are ascribed to the polar phospholipids existing in the SBL counterpart of each nanoliposome fraction. Whereas the fractions with larger zeta potential values specify that the representative fractions tend to repel each other and resist the agglomeration. This repelling phenomenon likely confirms that the larger the zeta potential is, the more likely the formulation is stable too. Largely, the zeta potential values ranging −30 mV and +30 mV are suitable for colloidal stability [40].

It is noteworthy to mention that the zeta potential values were reduced substantially in all test formulations after day 30. This reduction in zeta potential value was also recorded in the F6 (control) sample, thus considered time-specific. The highest zeta potential value was found for the F5 CEO formulation prepared using the lowest CEO concentration. This behavior was interesting but cannot be firmly associated with EOs’ concentration. The localization between the phenolics exists in the polar region of CEO and the opposing groups or the acyl chains abridged the repulsive forces among the phospholipid head groups [46]. Highly efficient encapsulation in the polymeric lattice can also be a significant solution to guarantee colloidal dispersion stability [47]. TTO-loaded nanoliposomes exhibit zeta potential values of −26.5 ± 1.44 mV. The representative zeta potential value increased at higher TTO concentrations in nanoliposomes. The zeta potential value was reduced to −7.01 ± 1.36 mV after day 30 and may imply agglomeration. Nevertheless, the zeta potential values for all TTO-loaded nanoliposomes (F1 to F5) were higher than those for the control sample (F6). The recorded zeta potential values of TTO-loaded nanoliposomes after day 1, day 7, and day 30 of storage are summarized in Table 4.

### 2.3. Topography and Surface Morphology Evalution

**Figure 1:**
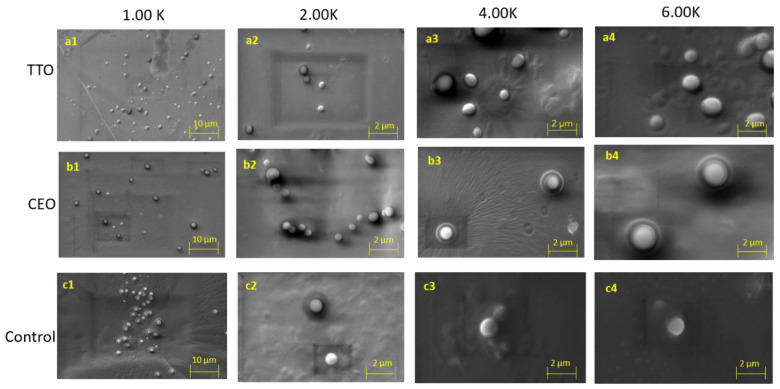
Representative scanning electron microscope images of TTO, CEO, and control samples (**a1**–**a4**) TTO, (**b1**–**b4**) CEO, and (**c1**–**c4**) control nanoliposomes at 1.0 K, 2.00 K, 4.00 K, and 6.00 K magnifications after storage at 4 °C.

SEM analysis was performed to visualize the topography and surface morphology of as-prepared EO-loaded nanoliposomes. Size distribution and morphology are listed as the most influential characteristics leading to the behavior of nanomaterials, which could be influenced during the drying process and crystal formation [38,48]. The nanomaterials’ deployment for drug delivery and biomedical applications relies on their pharmacokinetics and characteristics such as biocompatibility, stability, drug loading capacity, release time, and target efficiency [49]. In the present work, samples containing CEO and TTO-loaded nanoliposomes and control (empty vesicles) were recorded under 1.0 K, 2.0 K, 4.0 K, and 6.0 K magnification to analyze the morphology of nanoliposomes. The microscopic view of CEO, TTO, and control samples portrayed spherical shapes and uniform distribution (Figure 1). For the unloaded nanoliposomes, broad agglomeration zones related to the small particle size of this group of samples as confirmed by DLS can be observed (c1–c4). Nevertheless, for the series of TTO (Figure 1 a1–a4) and CEO (Figure 1 b1–b4), nanoliposomes exhibited less agglomeration, and a better distribution was observed, thus, indicating better stability compared to control samples [50]. Moreover, as confirmed by DLS data, it can be observed that the formation of clusters in control samples is due to more substantial interactions due to the smaller particle size compared to EO-loaded nanoliposomes. According to previous literature reports, these variations in size can be attributed to the loading of EOs into nanoliposomes [27,51]. Furthermore, TTO nanoliposomes exhibited fewer clusters than the CEO samples, also confirmed by the lower PdI values obtained by DLS analysis. Such aggregations as presented in the images may be associated with the lyophilization process applied during sample preparation [33].

### 2.4. ATR-FTIR Studies of CEO and TTO Nanoliposomes

**Figure 2:**
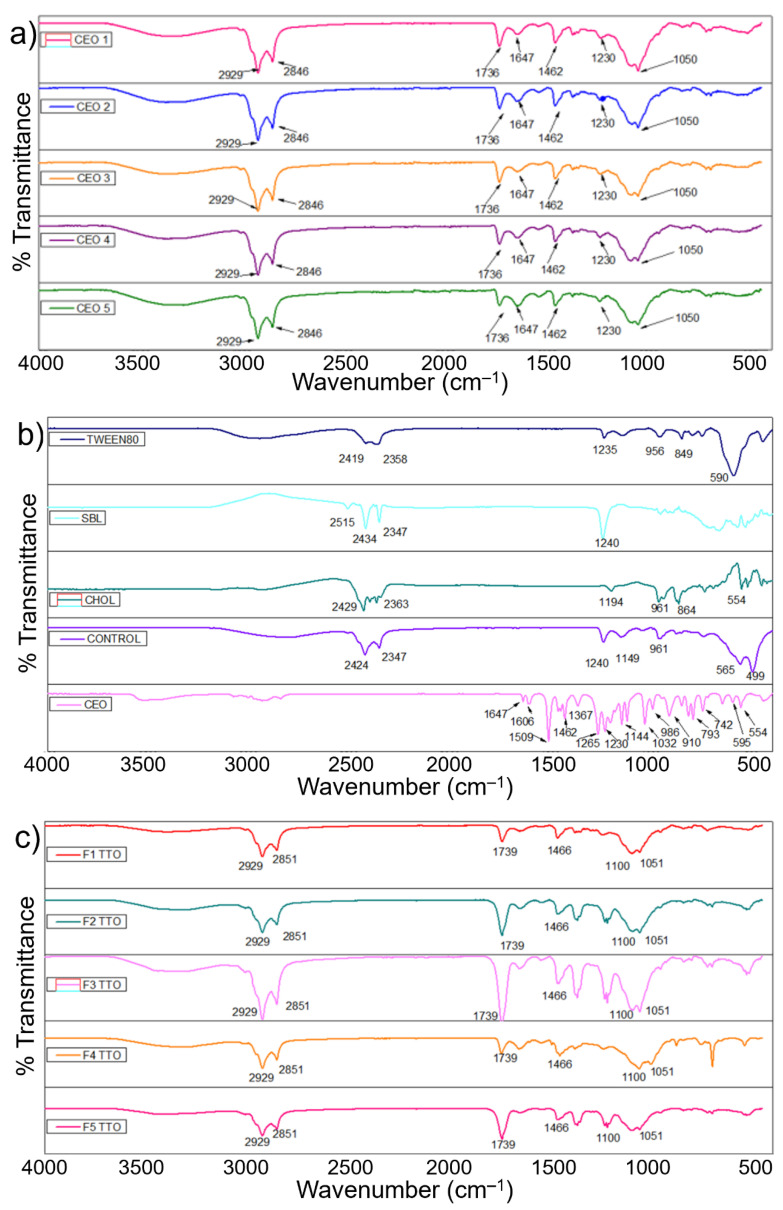

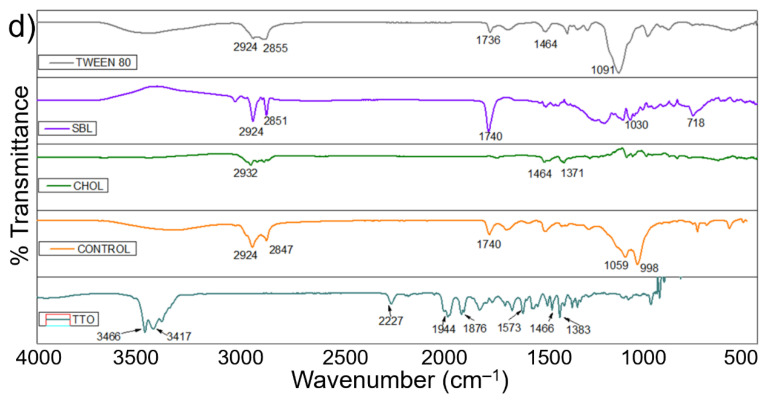
ATR-FTIR studies of CEO and TTO nanoliposomes (**a**) IR spectra of CEO nanoliposomes, (**b**) IR spectra of SBL, CHOL, Tween 80^®^, CEO and control, (**c**) IR spectra of TTO nanoliposomes, and (**d**) IR spectra of SBL, CHOL, Tween 80^®^, TTO, and control.

The characteristic ATR-FTIR peaks revealed the appearance of stretching alkane at 2929 cm^−1^, stretching aldehyde bands at 1736 cm^−1^, bending alkane bands at 1462 cm^−1^, and primary alcohol bands at 1050 cm^−1^ for CEO-loaded nanoliposome fractions. The representative peaks are shown in Figure 2a. The FTIR spectra of CEO unveiled peaks representing the presence of volatile compound frequencies [52]. The above-mentioned characteristic peaks also existed in the CEO-loaded nanoliposomes, demonstrating the existence of CEO in the matrix and confirming the EOs into nanoliposome fractions. The characteristic peaks that appeared at 961 cm^−1^ revealed bending alkene that may be related to CHOL and Tween 80^®^ bands (Figure 2b). Moreover, the presence of more intense peaks at higher concentrations of CEO can be attributed to the broad content of volatile groups in CEO or possible interaction between EOs and the lipid matrix [27].

In Figure 2c, the infrared spectra of TTO-loaded nanoliposomes showed characteristic bibrational bands related to the stretching alkanes at 2851 cm^−1^ and 1739 cm^−1^, stretching aldehyde bands at 1466 cm^−1^, bending alkanes related to the methylene group at 1100 cm^−1^, stretching of primary and secondary alcohol bnads at 1051cm^−1^. The peaks appeared at 1740 cm^−1^ (C=O), 1059 cm^−1^ (C-O), and 998 cm^−1^ (C=C), corresponding to stretching aldehyde, stretching of a primary alcohol, and monosubstituted bending alkene, respectively, as observed in Figure 2d. Moreover, strong peaks of TTO (1466 cm^−1^) were exhibited in nanoliposome samples but not in the control nanoliposome. Bands of phospholipids and excipients (2924 cm^−1^ and 2851 cm^−1^) were reflected in loaded and unloaded nanoliposomes, thus confirming the incorporation of EOs into the vesicles. These results confirm the null formation of new chemical compounds and interactions between precursor materials and EOs.

### 2.5. Entrapment Efficiency (EE%)

**Figure 3:**
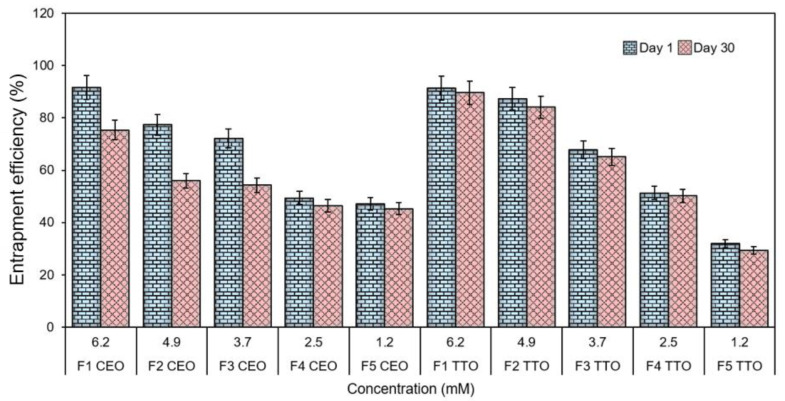
Percent EE of CEO and TTO nanoliposomes at day 1 and day 30 after preparation.

The CEO-loaded nanoliposome fractions were able to entrap up to 91.57 ± 2.5% of the respective EOs. Among TTO-loaded nanoliposome fractions, the highest amount of EOs entrapped was 91.4 ± 0.7%. It was detected that the EE% was influenced by the concentration of EOs, and the results are shown in Figure 3. Entrapment efficiency and vesicle size are critical parameters in a topical liposomal formulation. In an earlier study, Risaliti et al. [53] developed an *Artemisia annua* L. essential oil (AEO)-loaded nanoliposome and tested it for antifungal efficacy against resistant Candida strains. The encapsulation efficiency of 75% of EO-loaded liposomes was recorded over a 30-day storage period. Whereas, in this study, maximum entrapment efficiencies of up to 91.57 ± 2.5% and 91.4 ± 0.7% were obtained for CEO and TTO-based nanoliposomes.

### 2.6. Mycelial Growth Test—In-Vitro Antifungal Evaluation 

**Figure 4:**
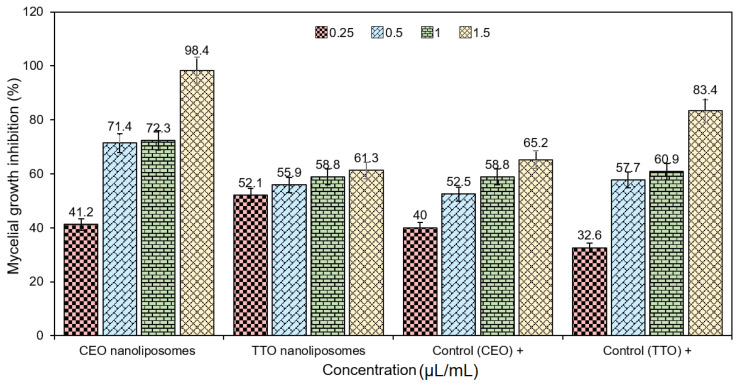
Anti-fungal potential of EO (CEO and TTO)-loaded active fractions against *T. rubrum* fungi.

The antimicrobial activity, at large, and antifungal activity, in particular, of various EOs is majorly due to the qualitative and quantitative characteristics of their biologically active entities, such as phenols (highest activity), alcohol, terpenes, and ketones [54]. CEO and TTO loaded fractions were tested against *T. rubrum* fungi. Comparative to TTO-loaded nanoliposomes, the CEO-loaded fractions had a higher MGI (%) (Table 5). The highest MGI (%) activities of CEO and TTO formulations were 98.4 ± 0.87% and 61.3 ± 0.6%, respectively, at 1.5 µL/mL concentration (Figure 4). These results show the efficacy of encapsulation of EOs into nanoliposomes to upsurge the anti-fungal activity against *T. rubrum* fungi. As per MGI (%) results, both formulations of EO nanoliposomes exhibited antifungal activity to a different extent. However, the performance of CEO nanoliposomes was better compared to TTO nanoliposomes. At the lowest concentration (0.25 µL/mL), TTO nanoliposomes presented higher inhibition than the positive control (TTO in bulk). Nevertheless, positive control of TTO was superior at concentrations of 0.5, 1, and 1.5 µL/mL, respectively. This behavior can be associated with the ability of TTO to disrupt the permeability of cell membrane structures at minimum inhibitory levels. On the other hand, a faster leakage at higher concentrations of EO from the nanoliposome matrix, this possibility is mainly related to volatile constituents in TTO [23].

## 3. Materials and Methods

### 3.1. Chemicals/Reagents/Software

Soybean lecithin was procured from a local market (Monterrey, Mexico). All other chemicals/reagents were of analytical laboratory grade with maximum purity and obtained from representative company suppliers. For instance, cholesterol (CHOL) was purchased from Sigma-Aldrich (Darmsdat, Germany). Two as used EOs, i.e., (1) TTO and CEO were procured from PACALI (Monterrey, Nuevo León, México). All other chemicals/reagents, e.g., Tween 80^®^, Chloroform, and Phosphate Buffer Saline (PBS), were of analytical laboratory-grade with maximum purity and were used as received without any further purification or treatment unless otherwise explained. 

### 3.2. In-House Development of TTO and CEO-Loaded Nanoliposomes

The EOs (TTO and CEO)-loaded nanoliposomes were freshly fabricated using a thin-film hydration–sonication method, as reported earlier [32,33,34]. For the said purpose, the mixed lipid components comprising SBL and CHOL in a 5:1 ratio were used to prepare 160-mL of stock-solution at 15 mM by dissolving in chloroform. Five different concentrations, i.e., 6.2, 4.9, 3.7, 2.5, and 1.2 mM of both EOs, each separately, were mixed with 10 mL of the above stock solution (SBL:CHOL, 5:1 ratio). The designations of nanoliposome formulations (F1 to F5) and control (F6) are summarized in Table 6.

**Figure 5:**
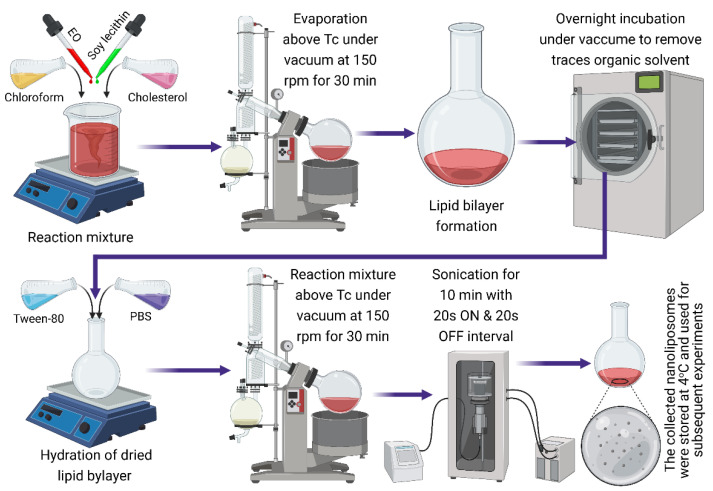
Illustration of as used thin-film hydration–sonication procedure to fabricate EO (CEO/TTO)-loaded nanoliposomes. Created with BioRender.com and extracted under premium membership.

Each formulation fraction was subjected to the rotary evaporator at 150 rpm and 60 °C. After 1 h in the rotary evaporator, the samples were moved to the desiccator for a 24 h period to remove the excess solvent traces. After the stipulated period of 24 h of incubation in the desiccator, the uniform thin lipid membranes were further hydrated for 30 min at 60 °C. Then, the above samples were subjected to sonication (sonicator probe—Branson ^®^ Sonifier SFX series) using a double-step 1/8” microtip (SFX550) at 60 °C and 120 Watts with 60% amplitude for 10 min. To avoid excessive heating during the sonication, around 20 s ON and 20 s OFF intervals were used to form small unilamellar TTO and CEO-loaded nanoliposomes. Finally, the resultant TTO and CEO-loaded nanoliposomes were stored at 4 °C for 30 days. All freshly prepared nanoliposome formulations were filtered using a 0.22 µm polytetrafluoroethylene syringe filter to obtain a uniform sample composition and subjected to characterization. Figure 5 illustrates a graphic depiction of the procedure used for the fabrication of EO-loaded nanoliposomes.

### 3.3. Characterization of TTO and CEO-Loaded Nanoliposomes

#### 3.3.1. Nano-Size Evaluation by DLS

A Zetasizer Nano ZS (Malvern Panalytical Instruments) was employed to record the average particle size distribution, zeta potential, and polydispersity index of each of the prepared nanoliposomes at 25 °C. All test samples were subjected to integrated dynamic light scattering (DLS) for a 60 s period. The MALVERN software was used to analyze the DLS data. Approximately 200 µL of freshly prepared samples were diluted (5:2) with a PBS solution and placed in a disposable polystyrene cuvette, DTS0012, to avoid multiple scattering. The diluted samples were then shifted to a Universal Dip Cell (ZEN 1002) to record the zeta potential values. Three different periods, i.e., day 1, day 7, and day 30, were chosen to perform the said analysis to check the stability of nanoliposomes (each prepared and tested in triplicate).

#### 3.3.2. Morphological Evaluation by SEM

All TTO and CEO-loaded nanoliposomes were subjected to the scanning electron microscope (SEM) (ZEISS EVO^®^ MA 25, Germany) at EHT: 15.00 kV to evaluate the morphological distribution. During sample preparation, each test nanoliposome was glazed with gold using a sputter coater under an Ar atmosphere (50 Pa) at 50 mA for 50 s. Finally, SEM images were taken at different magnifications. 

#### 3.3.3. Functional Attributes Evaluation by ATR-FTIR

The spectral evaluation of test nanoliposomes was executed using attenuated total reflection-Fourier transform infrared (ATR-FTIR) spectroscopy (Perkin Elmer, Beaconsfield, UK). The following specifications were used to record the spectral distribution, i.e., 4000 to 400 cm^−1^ wavenumber ranges, 16 scans, 2 cm^−1^ resolution, 0.50 cm/s speed, and 100 force gauge. For comparative purposes and a better understanding of the vibrational/stretching distributions, SBL, CHOL, and Tween 80^®^ were also scrutinized, each separately, to confirm the incorporation of TTO/CEO into nanoliposomes.

#### 3.3.4. Percent Entrapment Efficiency (%EE) Evaluation 

All test samples were subjected to ultracentrifugation (Optima XE 100, New York, NY, USA) with a Rotor SW32TI at 174,000× *g* for 3 h at 4 °C before calculating the %EE. The concentration of EO was measured spectrophotometrically UV/Vis at λ-max 298 nm. The following, Equation (1), was used to calculate the %EE:

(1) $$EE\left(\%\right)=\frac{Total\;amount\;of\;EO-Unentrapped\;EO}{Total\;amount\;of\;EO}\;\times \;100\%$$

### 3.4. Mycelial Growth Test—In-Vitro Antifungal Evaluation 

The antifungal activities of TTO and CEO-loaded nanoliposomes and control nanoliposomes were assessed by evaluating the contact effects on the mycelial growth of *T. rubrum*. Herein, an in-vitro procedure based on the agar dilution method was used, as reported earlier [35]. The fungal strain *T. rubrum* was re-cultured under standard fungal conditions. For the said purpose, the test EO-loaded nanoliposomes and control nanoliposomes, each separately, were disseminated in sterile PDA solution, which was additionally supplemented with 1% (*v*/*v*) Tween-80^®^. A 7-day-old fungal culture of *T. rubrum* was taken to prepare a 5 mm diameter disc from the culture edge and placed in the center of each Petri plate. All test samples were incubated at 25 °C for 3 days. Finally, the antifungal efficacy was accessed by measuring the MGI (%). The MGI (%) was calculated using Equation (2):

MGI (%) = [(*dc*−*dt*)/*dc*] × 100 (2)

where *dc* (cm) is equivalent to the mean colony diameter for the control sets and *dt* (cm) is equal to the colony diameter for the treatment sets. Each analysis was performed in triplicate.

## 4. Conclusions

In the present work, CEO and TTO-loaded fractions as nanoliposomes were fabricated and characterized to overawe the confines of EOs concerning their instability, high volatile nature, and potential as antifungal agents. At higher EO concentrations, an upsurge in the particle size was observed. This was mainly due to the interface between EOs with the lecithin bilayer’s acyl chains, which are liable for fluctuating the acyl chain order. The phospholipids and excipients utilized for the synthesis affected the vesicles’ surface charge and stability. An ATR-FTIR study confirmed EO bands’ presence in the nanoliposome formulations. These formulations exhibit the highest EE(%) values (91.57%) at 6.2 mM. Moreover, CEO and TTO nanoliposomes’ anti-fungal potential demonstrates their capability to inhibit mycelial growth at lower concentrations than the pure EOs against *T. rubrum* fungi.

## Figures and Tables

**Table 1:** The particle size distribution and PdI of CEO-loaded nanoliposomes (F1 to F5 fractions) and control sample (F6 fraction) after day 1, 7, and 30 of storage at 4 **°C**. Data were articulated as mean values ± standard deviation.

Sample Name	Concentration (mM)	Size Day 1 (nm)	PdI Day 1	Size Day 7 (nm)	PdI Day 7	Size Day 30 (nm)	PdI Day 30
F1 CEO	6.2	74.67 ± 0.81	0.43 ± 0.006	86.0 ± 41.43	0.488 ± 0.049	97.49 ± 1.48	0.483 ± 0.022
F2 CEO	4.9	72.88 ± 0.33	0.43 ± 0.002	68.26 ± 0.49	0.436 ± 0.003	120.63 ±1.40	0.320 ± 0.036
F3 CEO	3.7	71.43 ± 1.42	0.33 ± 0.040	71.45 ± 0.53	0.312 ± 0.003	102.5 ± 0.69	0.296 ± 0.008
F4 CEO	2.5	78.70 ± 0.66	0.42 ± 0.011	84.54 ± 0.54	0.307 ± 0.006	95.98 ± 0.61	0.313 ± 0.008
F5 CEO	1.2	76.39 ± 1.19	0.37 ± 0.051	76.09 ± 0.31	0.367 ± 0.045	129.23 ± 1.10	0.548 ± 0.008
F6 (Control)	0	32.43 ± 1.47	0.25 ± 0.002	105.10 ± 1.65	0.292 ± 0.007	110.83 ± 0.83	0.464 ± 0.003

**Table 2:** The particle size distribution and PdI of TTO-loaded nanoliposomes (F1 to F5 fractions) and control sample (F6 fraction) rations after day 1, 7, and 30 of storage at 4 °C**.** Data were articulated as mean values ± standard deviation.

Sample Name	Concentration (mM)	Size Day 1 (nm)	PdI Day 1	Size Day 7 (nm)	PdI Day 7	Size Day 30 (nm)	PdI Day 30
F1 TTO	6.2	44.84 ± 1.98	0.421 ± 0.007	37.27 ± 0.46	0.513 ± 0.012	88.54 ± 0.19	0.299 ± 0.006
F2 TTO	4.9	79.28 ± 1.51	0.429 ± 0.006	104.75 ± 0.21	0.456 ± 0.009	97.57 ± 1.58	0.33 ± 0.043
F3 TTO	3.7	93.51 ± 1.99	0.31 ± 0.005	81.45 ± 0.17	0.374 ± 0.004	102.47 ± 0.75	0.264 ± 0.006
F4 TTO	2.5	64.47 ± 1.82	0.440 ± 0.017	93.26 ± 0.41	0.493 ± 0.028	99.88 ± 0.38	0.277 ± 0.006
F5 TTO	1.2	37.12 ± 1.23	0.377 ± 0.007	92.69 ± 0.60	0.483 ± 0.007	95.99 ± 0.40	0.295 ± 0.006
F6 (Control)	0	32.43 ± 1.47	0.25 ± 0.002	105.10 ± 1.65	0.292 ± 0.007	110.83 ± 0.83	0.464 ± 0.003

**Table 3:** Zeta potential values of CEO nanoliposomes (F1 to F5 fractions) and control sample (F6 fraction) after day 1, 7, and 30 of storage at 4 °C. Data were articulated as mean values ± standard deviation.

Sample Name	Concentration (mM)	Zeta Potential Day 1 (mV)	Zeta Potential Day 7 (mV)	Zeta Potential Day 30 (mV)
F1 CEO	6.2	−28.21 ± 0.29	−25.14 ± 0.37	−8.24 ± 0.93
F2 CEO	4.9	−27.13 ± 0.33	−25.04 ±0.42	−8.15 ± 1.79
F3 CEO	3.7	−24.64 ± 0.32	−21.51 ±0.72	−7.64 ± 0.75
F4 CEO	2.5	−28.92 ± 0.45	−27.95 ± 0.74	−8.19 ± 1.07
F5 CEO	1.2	−30.39 ± 0.79	−29.43 ± 1.21	−8.39 ± 0.61
F6 (Control)	0	−27.54 ± 1.49	−20.5 ± 0.7	−8.66 ± 1.43

**Table 4:** Zeta potential values of TTO-loaded nanoliposomes (F1 to F5 fractions) and control sample (F6 fraction) after day 1, 7, and 30 of storage at 4 °C. Data were articulated as mean values ± standard deviation.

Sample Name	Concentration (mM)	Zeta Potential Day 1 (mV)	Zeta Potential Day 7 (mV)	Zeta Potential Day 30 (mV)
F1 TTO	6.2	−26.5 ± 1.44	−15.9 ± 2.28	−10.9 ± 0.98
F2 TTO	4.9	−24.2 ± 0.60	−12.01 ± 1.12	−7.01 ± 1.36
F3 TTO	3.7	−25.9 ± 0.65	−13.93 ± 0.79	−8.93 ± 1.20
F4 TTO	2.5	−21.1 ± 0.14	−15.80 ± 0.73	−7.80 ± 1.43
F5 TTO	1.2	−20.7 ± 0.58	−14.81 ± 0.71	−7.81 ± 1.09
F6 (Control)	0	−27.54 ± 1.49	−20.5 ± 0.7	−8.66 ± 1.43

**Table 5:** Mycelial growth inhibition (MGI) of CEO and TTO nanoliposomes. Pure CEO and TTO were positive controls and empty nanoliposomes were negative control. Data were articulated as mean values ± standard deviation.

Concentration (µL/mL)	MGI of CEO (%)	MGI of TTO (%)	Positive Control (TTO) (%)	Positive Control (CEO) + (%)	Negative Control (Empty Nanoliposome) (%)
0.25	41.2 ± 0.6	52.1 ± 0.4	32.6 ± 0.4	40 ± 0.4	0
0.5	71.4 ± 0.9	55.9 ± 0.2	57.7 ± 0.5	52.5 ± 0.5	0
1	72.3 ± 0.2	58.8 ± 0.5	60.9 ± 0.2	58.8 ± 0.3	0
1.5	98.4 ± 0.87	61.3 ± 0.6	83.4 ± 0.8	65.2 ± 0.1	0

**Table 6:** Processing conditions and designation of freshly fabricated nanoliposome formulations (i.e., F1 to F5) of EOs (CEO/TTO), and control (F6).

Component	Function	F1	F2	F3	F4	F5	F6 (Control)
Eos * (mM)	Bioactivesource	6.2	4.9	3.7	2.5	1.2	0
Soy lecithin (mg/mL)	Lipid	10	10	10	10	10	10
Tween 80^®^ (M)	Surfactant	0.076	0.076	0.076	0.076	0.076	0.076
Cholesterol (mg/mL)	Lipid	2	2	2	2	2	2
PBS (mL (0.03 M))	Saline buffer	20	20	20	20	20	20
Chloroform (mL)	Solvent	10	10	10	10	10	10

* Represent CEO/TTO in respective samples.

## Data Availability

All data related to this work is given here in the manuscript.
